# Papua New Guinea agri-food trade and household consumption trends point towards dietary change and increased overweight and obesity prevalence

**DOI:** 10.1186/s12992-021-00787-0

**Published:** 2021-11-27

**Authors:** Emily Schmidt, Peixun Fang

**Affiliations:** Development Strategy and Governance Division of the International Food Policy Research Institute (IFPRI), 1201 Eye St. NW, Washington, DC 20005 USA

**Keywords:** Papua New Guinea, Pacific, Agri-food trade, International trade, Sugar-sweetened beverages, Ultra-processed foods, Overweight, Obesity

## Abstract

**Background:**

Papua New Guinea (PNG) experienced positive GDP growth at approximately 4.3% per year during the last decade. With increases in overall wealth within the country, PNG is facing a double burden of malnutrition: comparatively high child stunting rates and a growing overweight and obesity epidemic. We focus on the latter by evaluating trends in agri-food import data from 2001 to 2018 and household consumption data from 2018 and 2009/10.

**Results:**

The analysis presented in this paper raises three red flags. First, international food import data suggest that the demand for ultra-processed, sugar-sweetened beverages and food have increased substantially over time in PNG. Sugar-sweetened beverages dominated the largest growth in processed food imports, increasing by 23% per capita per year between 2001 and 2018. Second, households across the country with a greater food expenditure on sugar-sweetened beverages have a higher probability of an overweight child (under 5 years old). Last, the probability of soft-drink consumption in PNG increases with greater income acquisition and improved market access. While the price of a soft drink is negatively correlated with the quantity consumed, analysis suggests that total household income has a quantitatively larger (and positive) association with soft drink consumption.

**Conclusions:**

Taxing (or increasing taxes on) sugar-sweetened beverages may not be a sufficient policy mechanism to curb overconsumption of soft drinks in PNG. Education and advocacy programs should be fostered that integrate improved dietary information on packaging, as well as greater access to and understanding of nutrition and diet information of common household consumption items. While increases in household income and market access are crucial to economic growth and transformation, PNG’s economic transition must be dovetailed with programs that expand and enhance health and nutrition information and education to improve household consumption decisions and overall household wellbeing.

**Supplementary Information:**

The online version contains supplementary material available at 10.1186/s12992-021-00787-0.

## Background

Pacific island nations comprise 9 of the top 10 countries in the world with the greatest obesity prevalence [33]. In addition, the double burden of malnutrition (defined by high rates of child stunting and high rates of overweight and obesity prevalence) in the Pacific Islands is challenging policymakers on both ends of the nutrition spectrum. In Papua New Guinea (PNG), 49% of children under 5 years old were stunted in 2009/10 (height for age below 2 standard deviations of the WHO growth standard median), while overweight prevalence among children under 5 years old was one of the highest in the region at 14% (WHO, [50]; PNG-HIES, 2009/10). Previous work has evaluated poverty and stunting prevalence in selected areas of PNG (Schmidt and Gilbert, 2019 [41];), however there has been very little analysis on recent overweight and obesity trends in the country.

PNG has experienced positive GDP growth at approximately 4.3% per year for the last decade, and more than 5% during the last 5 years (World Development Indicators, 2019). Research suggests that with greater productivity output (and associated higher household incomes) comes greater consumption demand for higher value food, particularly in developing countries [9, 20, 30, 35, 39]. Higher value foods can be both positive and negative from a nutrition standpoint. While demand for protein-dense foods (usually animal-sourced) increases with greater income (often filling a significant micro-nutrient gap in developing country diets), demand also increases for ultra-processed foods such as sugar-sweetened beverages and food, and pre-packaged meals containing higher saturated fat content. However, lack of recent data on household consumption and expenditure trends and robust anthropometric measurements in PNG make it near impossible to target policies and investments that promote specific nutrition and food security outcomes.

Several analyses have evaluated overweight and obesity prevalence in PNG (see for example, Pus et al. 2016 [37]), however there are no recent studies that evaluate the linkages of household consumption trends and child overweight / obesity prevalence. One of the most comprehensive evaluations of overweight and obese prevalence (among adults aged 15–64) in PNG was conducted more than a decade ago (2007/08) via the WHO STEPwise Approach to Surveillance of NCD Risk Factors (STEPS). Results found that approximately 39% of the adult population was overweight (32%) or obese (6.8%), however STEPS evaluation didn’t collect household food consumption data. The more recent PNG Demographic and Health Survey (2016–2018) collected child anthropometry data, however the results were not published due to inconsistency and error in data collection. Smaller studies have pointed to alarming rates of overweight and obesity in PNG in recent years. For example, Rarau et al. [38] sampled 785 adults in 3 provinces (Central, Eastern Highlands and Madang) in 2013–14 following the STEPS methodology (however the sample was not representative at any administrative level), and results suggest that 19 and 11% of respondents, respectively, were overweight or obese.

The increasing prevalence of obesity and overweight children and adults is not unique to Papua New Guinea.^1^ The United Nations highlighted the risk of overweight and obesity incidence in low and middle-income countries, committing resources to tackle prevalence of non-communicable disease such as diabetes and cardio-vascular disease associated with poor dietary patterns (United Nations, 2011 [48]). In the Pacific region alone, recent analysis reported that almost a quarter of adolescent age children are overweight, however statistics vary by country with Tonga and the Federated States of Micronesia representing some of the most acute cases whereby over half of the study sample was overweight [8, 34]. Kessarem et al. [25] found that 60% of students (grades 9–12) were overweight in Cook Islands, Niue, Tonga and American Samoa; authors linked overweight trends with lack of physical activity and daily consumption of carbonated sugar-sweetened beverages.

Assuming overweight and obesity trends follow other emerging market country experiences, not only individual health outcomes will increasingly deteriorate, but healthcare costs will continue to increase [23]. Jakovljevic et al. [24] warn that in more developed middle-income countries, out-of-pocket health spending on non-communicable diseases will become financially restrictive, putting a strain on household incomes and economic wellbeing.

Given the dearth of household food consumption data in PNG, we utilize agri-food trade import data from 2001 to 2018 from the Base pour l’Analyse du Commerce International (Database for International Trade Analysis in English, [3]; Guillaume and Zignago 2010 [16]) and a recent 2018 rural household consumption expenditure survey (IFPRI 2019 [22]) in tandem with the 2009/10 nationally representative Household Income Expenditure Survey (HIES) to examine household consumption trends and associated child overweight and obesity prevalence. A careful examination of agri-food import trends from 2001 to 2018 in PNG shows a significant increase in the imported value of ultra-processed food items, particularly sugar-sweetened beverages. Utilizing the 2009/10 HIES data, we find a strong association with child overweight and obesity incidence among households with greater sugar-sweetened beverage consumption, controlling for other household and geographic factors. Finally, we use the 2018 PNG Rural Household Survey on Food Systems (IFPRI 2019[22]) to explore the factors influencing the consumption of greater quantities of sugar-sweetened beverages to inform and better target policies and programs aimed at improving nutrition and consumption decisions among households in PNG.

The remainder of this paper is orghousehold consumption expenditure surveyanized into 5 sections. The following section evaluates changes in consumption demand over time using the BACI international trade database at the product level from 2001 through 2018. We provide a broad overview of agri-food imports, demonstrating that food imports are important to achieving food security and dietary diversity goals, however data trends show a shift towards greater consumption of less healthy ultra-processed food during the last 2 decades.  The Methods section describes the data and methodology used to evaluate the outcomes of increased consumption of ultra-processed foods on child overweight and obesity prevalence, and the household factors associated with increases in consumption of sugar-sweetened beverages.  The Results section discusses the outcomes of the probit model which suggests that greater sugar-sweetened beverage consumption increases the probability of child overweight prevalence. Further exploration of sugar-sweetened beverage consumption is then evaluated using a Heckman two-stage model to understand household correlates of increased soft drink consumption.  The Discussion section examines potential policy options and recommendations. Finally, we summarize results and discussion in the Conclusion section.

## Overview of PNG food imports

We disaggregate the BACI 6-digit trade database by food type to evaluate changes in agri-food trade, by average volumes and values over two five-year periods (2001–05 and 2014–18).^2^ Overall, agri-food imports comprised 16% of total imports between 2014 and 2018 and remain crucial to supporting food security and dietary diversity within PNG [42]. Rice and wheat (flour) combined comprised 24% of total agri-food imports between 2014 and 2018, with rice imports making up the largest share (16%) of overall agri-food import values (Table 1).

**Table 1:** Value of top 10 agri-food imports in 2001/05 and 2014/18 ($1000 Real 2016 USD)

Food imports	2001/05	2014/18	% of each item in total food import	% annual growth rate per capita (2001–2018)
Rice	68,970	111,086	16%	1.5%
Food preparations^b^	8947	74,761	11%	15.2%
Wheat and meslin	7421	56,989	8%	14.5%
Bottled waters (natural, sweetened, aerated)^a^	2642	54,119	8%	23.4%
Sheep or goat meat	32,063	52,332	7%	1.6%
Prepare or preserved fish	3312	33,577	5%	16.9%
Palm oil	4639	25,537	4%	11.6%
Poultry	705	22,424	3%	27.7%
Pasta (instant noodle)	992	15,875	2%	21.1%
Prepare or preserved meat (e.g. canned meat)	4378	14,637	2%	7.4%
Sub-total of top 10	134,067	461,338		7.6%
Total value of food imports	236,065	700,841		6.4%
Top 10 share of total food imports	57%	66%		

**Source:** Authors’ calculations using BACI International trade database at the product level

**Note**: ^a^Bottled water includes flavored and sweetened water, and other non-alcoholic beverages such as soda, but does not include fruit juice. ^b^ Food preparations are comprised of processed food items including: protein concentrates, products derived from dried milk, butter substitutes and syrups

Growth in animal sourced agri-food imports during the last two decades (5% per capita per year from 2001 to 2018) reflects a shift in demand towards increased consumption of protein-dense foods. This is a positive transition given that PNG has a very small domestic livestock sector, coupled with low levels of protein consumption across poor and non-poor households [41].^3^ Poultry imports increased, from a low base, more than 30-fold (Real 2016 USD) on average between 2001 and 05 and 2014–18, growing at 34% per capita per year (Table 1). Preserved and prepared fish imports also increased substantially with Thailand comprising the largest share of demand in the form of tinned mackerel for domestic consumption, which is less expensive than the tinned tuna that PNG directs towards the export market.

Agri-food imports in PNG have broadened and increased the access to greater dietary diversity and protein dense foods. However, agri-food import data also suggest a rapid growth in the total import value of processed and ultra-processed foods. This impressive growth is particularly concerning given the growing evidence linking these foods with poor diets and adverse health outcomes including greater risk of cardiovascular disease, hypertension, and cancer [4, 5, 10]. Levy et al. [27] and Srour et al. [46] demonstrate through clinical trials that greater consumption of ultra-processed foods are associated with higher risk of Type 2 diabetes.

To further explore increases in processed food demand in PNG, we disaggregate agri-food imports into 4 categories following Monteiro [31] that uses the NOVA classification system to classify agri-food process levels: 1) minimal processed or unprocessed; 2) processed culinary ingredients (e.g. oil, sugars, etc.); 3) processed food (e.g. preserved vegetable/fruit/fish/meat); and 4) ultra-processed foods (e.g. pasta, sausages, sugar-sweetened beverages). Minimally processed foods in level 1 are treated to ensure stability for transport. For this analysis, we consider processed food imports to include levels 2–4. Classifying each agri-food import type by processing category suggests that processed foods (levels 2–4 in Table 2) accounted for 49% of total agri-food imports on average between 2014 and 2018 in Papua New Guinea (Table 2). Overall, processed agri-food imports increased by 11% per capita per year from 2001 to 2018. Ultra-processed imports comprised almost 70% of processed imports (34% of total food imports) and increased by 12% per capita per year from 2001 to 2018, representing the largest growth among processed goods, albeit from a low base (Table 2).

**Table 2:** Imported processed foods by process level ($1000 Real 2016 USD)

Processed food level	2001/05	2014/18	% annual growth rate per capita (2001–2018)	% of total processed (level 2–4) import value in 2014/18
1) Minimal processed / unprocessed	166,921	354,544	3.7%	
2) Processed culinary ingredients	13,079	48,506	8.2%	14%
3) Processed food	13,629	56,282	9.1%	16%
4) Ultra-processed food	42,435	241,508	11.8%	70%
Total processed (levels 2–4)	69,144	346,296	10.7%	100%
Total food imports	236,065	700,841	6.4%	
% of processed food in total imports	29%	49%		

**Source:** Authors’ calculations using BACI International trade database at the product level

**Fig. 1:**
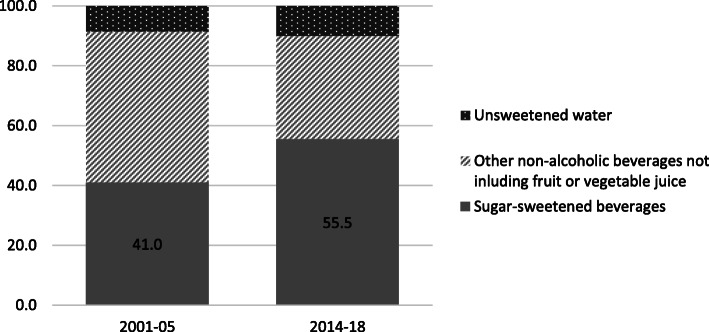
Share of sugar-sweetened beverage value in non-alcoholic drink imports (2001–05 and 2014–18). Source:Authors’ calculations using BACI International trade database at the product level

The largest growth in processed foods in terms of value imported occurred in non-alcoholic drinks which is the second highest value processed import after food preparation ingredients. Non-alcoholic drinks grew, on average, 23% per capita per year between 2001 and 18 (Table 3). Within the non-alcoholic drink category, sugar-sweetened beverages (i.e. sweetened water, cola, etc.) which comprises over half of the value of non-alcoholic beverage imports, experienced the largest growth. While in 2001–05, sugar-sweetened beverages comprised about 41% of the value of non-alcoholic beverage imports, by 2014–18, sugar-sweetened beverages comprised 56% of the value of non-alcoholic drinks, and 9% of total processed food imports (Fig. 1).

**Table 3:** Imported processed foods (level 2–4) by food type ($1000 Real 2016 USD)

Food group	2001/05	2014/18	% annual growth rate per capita (2001–18)	% of processed in total agri-food import within food group 2014/18	% of total processed import value in 2014/18
Food preparations^a^	8947	74,761	15.2%	100%	22%
Non-alcohol drinks	2642	54,119	23.4%	91%	16%
Oil and fats	16,028	43,167	5.6%	73%	12%
Others^b^	10,946	36,302	7.3%	82%	10%
Fish and seafood	3355	33,838	16.9%	20%	10%
Grain (pasta, bread, etc)	3524	31,038	15.7%	14%	9%
Sweetener	4592	25,319	11.6%	99%	7%
Animal meat	4508	16,235	8.0%	9%	5%
Dairy	7634	13,068	2.0%	67%	4%
Vegetable & legumes	3228	9893	6.6%	62%	3%
Fruit & nuts	3741	8555	4.3%	65%	2%
Total agri-food processed imports	69,144	346,296	10.7%		100%
Total agri-food imports	236,065	700,841	6.4%		
Share of processed imports	29%	49%			

**Source:** Authors’ calculations using BACI International trade database at the product level

**Note:**^a^ Food preparations are categorized in a 6-digit code that cannot be disaggregated into respective food groups. It includes items such as: protein concentrates, products derived from dried milk, butter substitutes and syrups. ^b^Others includes malt extract products, concentrates of tea or coffee, sauces, yeasts, etc.

To compare value growth between different ultra-processed food groups, we disaggregate imports into sugar-sweetened foods, sugar-sweetened beverages, high-saturated fat foods, and other ultra-processed foods (see Appendix Table 1 for a list of each ultra-processed food within each category).^4^ The value of sugar-sweetened food, sugar-sweetened beverages, high saturated fat food, and other ultra-processed food imports increased substantially by 9, 12, 9, and 8% per capita per year, respectively. The share of the ultra-processed food in total food imports increased from 22 to about 36% on average between 2001-03 and 2016–18 (Table 4).

**Table 4:** Value of ultra-processed food imports from 2001 to 18 ($1000 Real 2016 USD)

Period	Less-healthy ultra-processed* food imports				Share of ultra-processed food imports in total food imports
	Sugar-sweetened food	Sugar-sweetened beverage	High-saturated fat food	Other ultra-processed	
2001–03	6023	9510	23,860	7650	22%
2004–06	9125	16,455	25,821	10,294	23%
2007–09	14,361	23,570	37,513	15,729	22%
2010–12	26,127	63,648	78,113	38,785	26%
2013–15	29,918	89,579	123,719	44,186	38%
2016–18	29,036	65,522	117,231	31,731	36%
% annual growth rate per capita(2001–18)	9.0%	11.6%	9.1%	7.9%	

**Source:** Authors’ calculations using BACI International trade database at the product level

**Note**: ^*^ A few items, which are not ultra-processed (e.g. butter, cheese, and sweetened milk), are included to reflect sugar-sweetened food and beverage, and high-saturated fat food groups

Comparing PNG’s ultra-processed food imports to other countries in the Pacific, using the BACI trade database and the same food category types (detailed in Appendix Table 1) shows that PNG is the 5th largest importer, in terms of value of ultra-processed foods in the region (Table 5). Overall, approximately 37% of the value of PNG’s processed agri-food imports were ultra-processed between 2014 and 2018.^5^ Within the ultra-processed agri-food group, PNG dedicates a substantially larger share of the value of ultra-processed imports to sugar-sweetened beverages (28%) compared to most countries with high shares of ultra-processed food imports in the region.

**Table 5:** Share of less-healthy food groups in total ultra-processed foods (2014–2018)

Country	Share of ultra-processed foods in total food imports	Share of food type within ultra-processed* food imports			
		Sugar-sweetened food	Sugar-sweetened beverage	High-saturated fat	Other ultra-processed
Marshall Islands	50%	3%	14%	68%	15%
French Polynesia	47%	14%	13%	62%	12%
Vanuatu	46%	8%	20%	56%	16%
Tonga	40%	6%	19%	56%	19%
**Papua New Guinea**	**37%**	**11%**	**28%**	**47%**	**13%**
Kiribati	34%	6%	23%	45%	26%
Micronesia	34%	4%	16%	59%	21%
Philippines	33%	16%	33%	41%	10%
East Timor	33%	6%	40%	24%	29%
Samoa	32%	7%	22%	42%	29%
Solomon Islands	29%	8%	22%	37%	33%
Fiji	23%	13%	32%	41%	13%
Malaysia	22%	22%	29%	37%	12%
Indonesia	20%	16%	40%	37%	7%

**Source:** Authors’ calculations using BACI International trade database at the product level

**Note:** A few items, which are not ultra-processed (e.g. butter, cheese, and sweetened milk), are included to reflect sugar-sweetened food and beverage, and high-saturated fat food groups

The significant increase of imported sugar-sweetened food and beverage items in PNG begs the question of whether there are trends in the household consumption data that link reported consumption of these food items to overweight or obesity prevalence among household members. The following section describes the data and methodology used to explore consumption trends and obesity prevalence, and related analysis aimed at understanding household characteristics associated with increased consumption of sugar-sweetened beverages.

## Methods

We utilize the most recent, comprehensive data sources on consumption and anthropometry available in PNG. While the Household Income Expenditure Survey (HIES) 2009/10 is dated, it is the only nationally representative data available to calculate detailed child overweight and obesity prevalence and associated household food consumption. To explore more recent household consumption trends, we use the 2018 IFPRI Rural Household Survey on Food Systems (RSFS) to evaluate household characteristics that lead to increased soft drink consumption.^6^ Given the limited availability of processed food options in rural areas, soft drink consumption provides a good indicator of demand for sugar-sweetened beverages given that many small trade shops in rural areas of PNG have soda available for sale. In addition, soft drinks accounted for 56% of all sugar-sweetened beverage expenditure in the HIES 2009/10 data.

### Household income expenditure survey (HIES) 2009/10

The HIES 2009/10 collected data on household food consumption via a detailed survey module that asked each survey household to keep a food diary of every food item and quantity that the household consumed each day over a two-week period. The HIES also collected anthropometry data of children under 5 years old. Using these two pieces of information, we identify: 1) which households have at least one overweight or obese child (using the World Health Organization (WHO) child growth standards) 7, and 2) potential associations of child overweight prevalence with household consumption profiles of specific food types.

We evaluate consumption patterns within each household using the same NOVA classification system (Monteiro, 2019) described in the trade analysis above. Similarly, we further disaggregate food items into ultra-processed categories (sugar-sweetened food, sugar-sweetened beverages, and high-saturated fat food) to evaluate whether consumption of these ultra-processed food types is associated with child overweight or obesity prevalence within the household. Next, we employ a probit model which has a binary response variable *Y* that takes the value of 1 if the household (*i*) has at least one overweight child as a household member, and 0 if there are no overweight children living in the household. We hypothesize that a vector of *X* household characteristics influence the outcome *Y* on household *i*, in which the model takes the form:

1 $$ \Pr \left[{Y}_i=1|X\right]=\Phi \left({X}_i^{\hbox{'}}\beta \right), $$

where *Pr* denotes probability, and Φ is the Cumulative Distribution Function (CDF) of the standard normal distribution. The parameters *β* are estimated by maximum likelihood.

We assume other household characteristics, in addition to household expenditure levels of ultra-processed food types, may influence child overweight prevalence. Included in the *X* vector of household characteristics is: total household expenditure (used as a household income proxy), household size, sex of the household head and household head education level. We hypothesize that households with higher incomes would have more flexibility to buy a greater amount and diversity of consumption items, and over-consumption may lead to overweight or obesity prevalence. Household head education level may affect consumption choices related with income-earning opportunities, but could also affect consumption patterns depending on whether a household head received nutrition and food preparation education that included healthy eating choices. We include household size and the sex of the household head to control for factors related to household decision-making and attention to childcare and wellness. While we do not have detailed information on daily exercise or individual labor profiles, we include whether a household is in a metro (Lae or Port Moresby), urban, or rural area, recognizing that different types of labor (often geographically defined) require different levels of calorie intake. In addition, urban households would have greater access to markets and processed food items. Finally, we control for administrative region to account for region specific differences and food preferences.

### IFPRI rural survey on food systems

The results of the probit model suggest that households that consume more sugar-sweetened beverages are more likely to have at least one overweight or obese child (detailed analysis is discussed in the results section). Thus, we utilize the more recent 2018 IFPRI Rural Survey on Food Systems (RSFS) to identify household level characteristics that are associated with increased soft-drink consumption (Appendix Table 6 reports a similar analysis using the HIES 2009/2010 data for comparison).

In doing so, we employ Heckman’s two-stage model to estimate the marginal effects of hypothesized household characteristics (household income, the reported price of a soft drink, access to a market, household size, household head sex and education level, and number of children) on household level soft-drink consumption (Heckman, 1979 [18]).^8^ Approximately 40% of households in the 2018 PNG-RSFS reported consuming a soft drink during the 1 month recall period of the survey questionnaire. Given the rural focus of the household survey, we assume that the majority of zero soft-drink expenditures are due to household inability to purchase soft-drinks because they are either cost-prohibitive via direct costs (available soft-drink price) or indirect costs related to market access. Under this hypothesis, we assume that non-consuming households may have a latent demand for soft drinks.

We only observe household soft-drink demand when it is positive. Heckman (1979 [18]) developed a two-stage estimator to mitigate the potential sample selection bias that arises from a nonrandom sample of non-zero, positive values. For the case presented here, stage one of the Heckman two-stage estimator employs a probit model that determines the probability that a given household purchases a soft drink for home consumption. Predicted values from the first stage probit equation are used to estimate an inverse Mills ratio for each observation, which is then used as an instrument in the second stage.

The desired consumption (soft drink expenditure per capita per year) equation which causes sample selection, is specified as

1 $$ {C}_j^{\ast }={\gamma}^{\hbox{'}}{z}_j+{u}_j $$

where ${C}_j^{\ast }$ is per capita household consumption of soft-drinks, *z*_*j*_ is a vector of variables associated with soft-drink consumption, which is only observed if the household reported drinking at least one soft-drink, and *u*_*j*_ is the error term with a bivariate normal distribution with zero mean. The variable ${C}_j^{\ast }$ is not observed, but households report whether they consumed a soft-drink or not, so that

1 $$ {C}_j^{\ast }=1\ \mathrm{if}\ {C}_j^{\ast }>0 $$

and

$$ {C}_j^{\ast }=0\ \mathrm{if}\ {C}_j^{\ast}\le 0 $$

Let *w*_*j*_ represent the total expenditure per capita of soft drinks per household:

2 $$ {w}_j={\beta}^{\hbox{'}}{x}_j+{\varepsilon}_j, $$

whereby, *x*_*j*_ is the vector of variables affecting per capita household soft-drink consumption and *ε*_*j*_ is the error term with a bivariate normal distribution with zero mean. Under the assumption that the error terms are jointly normal, the second stage linear regression is defined as

$$ E\left({W}_j|{C}_j=1\right)=E\left({W}_j|{C}_j^{\ast }>0\right)=E\left({W}_j|{u}_i>-{\gamma}^{\hbox{'}}{z}_j\right) $$

$$ ={\beta}^{\hbox{'}}{x}_j+E\left({\varepsilon}_j|{u}_j>-{\gamma}^{\hbox{'}}{z}_j\right) $$

3 $$ ={\beta}^{\hbox{'}}{x}_j+{\rho \sigma}_{\varepsilon }{\lambda}_j\left({\alpha}_u\right) $$

To correct for potential sample bias, the ordinary least squares (OLS) regression represented in eq. 3 includes a vector of independent variables *x* and the inverse Mills ratio *λ*_*j*_(*α*_*u*_) as regressors to estimate *β*. *ρ* is the correlation between the error terms (i.e. between unobserved determinants of the probability of consuming a soft-drink, *u*, and the unobserved determinants of the yearly per capita soft-drink expenditure in the household (*ε*)), and *σ* is the standard deviation of the error term *ε*.

## Results

### Factors associated with child overweight prevalence: HIES (2009/10)

The HIES 2009/10 analysis utilizes the subsample of households that reported a child under 5 years old with complete anthropometry data (1721 out of 4191 households had at least one child with complete anthropometric data and sufficient household consumption and expenditure data). Approximately 15% of households with children under 5 years old had at least one child that was overweight (8% of households) or obese (7% of households).

Descriptive statistics suggest that sugar-sweetened beverage consumption was significantly more prevalent among households with at least one overweight (overweight includes obese) or obese child (Table 6). However, there are no significant differences between households with and without an overweight or obese child in consumption of sugar-sweetened food, high-saturated fat food or other ultra-processed foods. Disaggregating households by poor (bottom 40% of expenditure distribution) and non-poor status, consumption of both sugar-sweetened food and beverage is higher in households with at least one overweight or obese child. Among poor households, consumption of sugar-sweetened beverages is significantly higher in households with at least one obese child (with weaker significance for overweight prevalence).^9^

**Table 6:** Share of food expenditure on ultra-processed goods of households with at least one overweight or obese child

Share of total food expenditure	Not obese	Obese	t-test	Not overweight	Overweight^a^	t-test
*Panel A: All households with children under 5 years old*						
All ultra-processed food	7.0%	8.2%		6.9%	8.0%	*
Sugar-sweetened food	0.5%	0.7%		0.5%	0.6%	
Sugar-sweetened beverage	2.5%	3.4%	**	2.4%	3.3%	***
High saturated fat food	3.0%	3.1%		3.0%	2.9%	
Other ultra-processed food	1.0%	1.1%		1.0%	1.2%	
Processed food	5.4%	6.6%	**	5.3%	6.1%	*
Processed culinary ingredients	4.6%	7.5%	***	4.5%	6.2%	***
Minimally or unprocessed	83.1%	77.8%	***	83.3%	79.7%	***
*Sample size*	*1601*	*121*		*1459*	*263*	
*Panel B: Non-poor (top 60% expenditure distribution)*						
All ultra-processed food	8.2%	8.9%		7.9%	9.7%	*
Sugar-sweetened food	0.6%	1.2%	***	0.6%	0.9%	**
Sugar-sweetened beverage	3.1%	3.5%		2.9%	4.3%	***
High saturated fat food	3.6%	3.2%		3.5%	3.5%	
Other ultra-processed food	0.9%	1.0%		0.9%	0.9%	
Processed food	5.6%	5.5%		5.5%	6.0%	
Processed culinary ingredients	4.4%	5.0%		4.4%	5.0%	
Minimally or unprocessed	81.9%	80.6%		82.2%	79.3%	**
*Sample size*	*851*	*67*		*785*	*133*	
*Panel C: Poor (bottom 40% expenditure distribution)*						
All ultra-processed food	5.7%	7.6%	*	5.7%	6.5%	
Sugar-sweetened food	0.4%	0.2%		0.4%	0.3%	
Sugar-sweetened beverage	1.8%	3.2%	***	1.8%	2.5%	*
High saturated fat food	2.4%	3.0%		2.4%	2.4%	
Other ultra-processed food	1.1%	1.2%		1.0%	1.4%	
Processed food	5.2%	7.5%	***	5.2%	6.2%	
Processed culinary ingredients	4.8%	9.5%	***	4.7%	7.2%	***
Minimally or unprocessed	84.4%	75.3%	***	84.5%	80.0%	***
*Sample size*	*750*	*54*		*674*	*130*	

Source: Authors’ calculations using HIES 2009/10

Note: 81 (2.7%) children observations from 79 households were dropped because their height or weight were 3 standard deviation below or above the median value of the same age group, an additional 216 (7.4%) children observations from 194 households were dropped because of biologically implausible weight-for-height z-scores (i.e. z > 5 or z < −5). ^a^Overweight includes obese

Recognizing that other influencing factors may shape child anthropometric outcomes, we use a probit regression to better evaluate how the consumption of different food types is associated with the probability of a household with an overweight or obese child. Descriptive statistics of the model regressors are reported in Appendix Table 2. We run two probit models using different samples from the HIES 2009/10. The first model uses the national sample that includes 1721 households from all regions of the country. The second model focuses on households in Momase region and includes 545 households. We focus on Momase region to provide a parallel analysis with the 2018 RSFS survey data where ¾ of the survey sample was located in different provinces of Momase region.^10^

The marginal effects from the probit model are reported in Table 7 (Probit model estimates are reported in Appendix Table 3). Results suggest that a greater expenditure share of sugar-sweetened beverages in total household food purchases increases the probability of a child being overweight (which includes obese), controlling for other potential household influencing factors. 11 Specifically, a 1% increase in the expenditure share of sugar-sweetened beverages increases the probability of a child being overweight by 0.5%. For Momase region, the estimated effect is stronger, suggesting that a 1% increase of sugar-sweetened beverages expenditure share increases the probability of a child being overweight by 1%. While greater consumption of sugar-sweetened beverages has a significant positive effect on child overweight prevalence, we find no significant association when focusing on correlates of obesity prevalence. This may be due to the fact that the survey data only collected anthropometry data for children under 5 years old, resulting in a small sample of obese children in this age category. In addition, effects of increased sugar-sweetened beverages on child obesity may require a longer time horizon than the first 5 years of life.

**Table 7:** Marginal effects of household characteristics associated with an obese/overweight child (under 5)

Dependent variable: Household with at least one obese/overweight child (under 5)	Obese child		Overweight child^**a**^	
	All regions	Momase region	All regions	Momase region
Share of consumption of sugar-sweetened food in total food (%)	0.228	0.294	0.101	0.935
	(0.497)	(0.495)	(0.648)	(0.754)
Share of consumption of sugar-sweetened beverages in total food (%)	0.173	0.684	0.508**	1.091**
	(0.203)	(0.424)	(0.222)	(0.507)
Share of consumption of high-saturated fat food in total food (%)	0.063	−0.404	0.074	− 0.011
	(0.170)	(0.312)	(0.242)	(0.324)
Total HH expenditure, thousands (PGK/capita/year)	−0.002	0.002	−0.010	− 0.003
	(0.005)	(0.004)	(0.008)	(0.007)
Household in metro area (0/1)	0.031	0.019	0.025	0.015
	(0.035)	(0.034)	(0.038)	(0.049)
Household in urban area (0/1)	−0.030	− 0.031	− 0.038	− 0.074
	(0.020)	(0.045)	(0.026)	(0.060)
Number of children (0–15)	0.001	0.006	0.010	0.028***
	(0.007)	(0.008)	(0.009)	(0.010)
Household size	0.006	0.006	0.012**	0.004
	(0.005)	(0.005)	(0.005)	(0.008)
Female Household head (0/1)	−0.017	− 0.003	0.034	−0.084
	(0.028)	(0.046)	(0.053)	(0.070)
Age of Household head	−0.001	− 0.001	− 0.002*	−0.000
	(0.001)	(0.001)	(0.001)	(0.001)
Household head completed primary education (0/1)	0.007	−0.021	0.027	−0.018
	(0.019)	(0.030)	(0.022)	(0.047)
Highland region (base = Southern)	0.089***		0.198***	
	(0.031)		(0.038)	
Momase region (base = Southern)	−0.004		−0.014	
	(0.024)		(0.028)	
Island region (base = Southern)	0.016		−0.015	
	(0.034)		(0.047)	
Pseudo R2	0.045	0.082	0.086	0.089
N Observations	1721	544	1721	544

^a^Overweight sample includes obese

Note: Average marginal effect is reported. Robust standard errors clustered by census unit in parentheses. *** *p* < 0.01. ** *p* < 0.05. * *p* < 0.1

### Factors associated with increases in soft drink consumption: RSFS (2018)

Given that there are no updated, nationally representative consumption and expenditure data in PNG since 2009/10, we utilize the IFPRI Rural Household Survey on Food Systems (RSFS) to evaluate more current household consumption trends. The RSFS collected comprehensive data on household consumption and expenditure, and anthropometry for children under 5 years of age, however several challenges should be noted when interpreting the data. First, there are very few observations of overweight or obese children given the rural focus of the IFPRI survey. Thus, we are unable to evaluate food consumption correlates of childhood overweight or obesity prevalence. Second, given that the RSFS survey focused on rural household consumption trends, less detail of urban processed food consumption is available in the data. Thus, we use information on reported household consumption of soft drinks as a proxy indicator to identify household characteristics that are associated with greater consumption of sugar-sweetened beverages. Finally, the RSFS sample was located in Momase region, and included 3 geographically diverse provinces (East and West Sepik, and Madang) and Southern Bougainville.

Data collection of the RSFS survey spanned 3 months (May–July, 2018) and collected weekly and monthly consumption and expenditure data of a detailed list of food items. Households were asked whether they had purchased soft drinks in the last month, and if so, the price and quantity of soft drinks consumed in the household during that month. In total, 1026 households were surveyed in 4 lowland provinces of PNG. This analysis utilizes 1023 observations that have complete information of the relevant variables used in the Heckman two-stage model (Appendix Table 4).

Focusing on the first stage probit equation of the two-stage Heckman model, we select household characteristics that may affect participation (whether or not a household consumed a soft drink in the past month). We hypothesize that access to a market or trade shop that sells soft drinks is an important indicator of participation. The RSFS survey focused on rural households where walking was a primary means of mobility (the predominant mode of transportation of the Madang sample was via canoe across the Ramu river and associated tributaries), thus sex and age of the household head may affect access to and frequency of market interactions. During community focus groups, respondents identified extended travel times, insecure transportation routes and inadequate infrastructure as primary obstacles to market access. We also assume that total household expenditure, educational attainment of the household head, and the price of a soft drink would affect household consumption via household income and opportunity costs. Relatedly, brand marketing of soft drinks is aimed towards younger individuals, which may affect consumption demand differently depending on the age of the household head. Assuming that consumption is somewhat evenly divided within the household and among children, we include household size and number of children under 15 years of age as potential characteristics that may affect consumption decisions. Finally, we include a set of province fixed effects to control for differences in geographic and social characteristics and consumption preferences.

In the second stage equation (evaluating factors associated with the quantity per capita of soft-drink consumption), total household expenditure (proxy variable for household income) and unit price of a soft drink are the principal covariates we evaluate for this analysis. However, we also maintain household size in the second stage to account for potential factors influencing volume purchase per capita. Following the Heckman two-stage model specification, an exclusion restriction is required whereby at least one variable which appears in the first stage probit (participation) equation is absent in the second stage (level) equation. We assume that distance to a market is associated with whether a household purchases a soft drink, but once that decision is made, distance does not directly influence the amount purchased or consumed. Additional exclusions in the second stage equation include the characteristics of the household head (sex and age). We also assume that years of education of the household head and number of children within the household may be associated with whether to purchase a soft drink (weighing the trade-offs of purchasing a less healthy drink versus another beverage), but not on the amount purchased or consumed. We test these exclusion assumptions and results show that adding these covariates iteratively to the second stage regression has little effect on overall results (sensitivity results to the exclusion restriction are provided in Appendix Table 5).

Table 8 reports the coefficients for the first stage probit equation and the second stage levels equation. We report both the coefficients for the levels equation estimated without correcting for selection bias (column B), and estimated by Heckman’s procedure (Column C). The coefficients in column B only evaluate households that reported purchasing and consuming at least one soft drink (412 households). Correcting for selection bias via the Heckman procedure (column C) suggests that the factors influencing households’ decision to consume a soft drink may differ from the factors that condition how much soft drink to consume, which is also reflected in the coefficient of the inverse Mills ratio. Conditional and unconditional marginal effects calculated at the mean are presented in columns D and E, respectively. The conditional marginal effect reports the effect of a covariate among consumers (those that reported purchasing a soft drink). The unconditional marginal effect considers the increased probability of purchasing a soft drink and potential sample selection bias using the Heckman two-stage estimator.

**Table 8:** Heckman sample selection model for soft drink expenditure per capita

Dependent variables: Expenditure on soft drink per capita per year	Participation equation (Probit)	Consumption equation		Marginal effects	
		Without correction^b^	Heckman procedure	Conditionalmarginal effects	Unconditionalmarginal effects
	A	B	C	D	E
Log of total household expenditure (PGK/capita/year)	0.676***	23.976***	12.477*	24.499***	19.539***
	(0.077)	(3.123)	(7.112)	(7.247)	(2.429)
Household-level unit soft drink price (PGK/Liter)	−0.461***	4.036**	8.784***	0.583	−6.708***
	(0.082)	(1.641)	(3.215)	(3.526)	(2.435)
Household-level unit soft drink price squared (PGK/kg)	0.024***	−0.066	−0.300*	0.124	0.405***
	(0.004)	(0.092)	(0.166)	(0.182)	(0.128)
Euclidean distance to major market town^a^ (km)	−0.014**			− 0.243**	−0.299*
	(0.007)			(0.121)	(0.167)
Euclidean distance to major market town squared (km^2^)	0.000			0.001	0.001
	(0.000)			(0.001)	(0.001)
Household size	0.069**	−1.519*	−2.933**	−1.714	0.375
	(0.033)	(0.907)	(1.228)	(1.358)	(0.801)
Household head completed primary education (0/1)	0.076			1.359	1.669
	(0.124)			(2.208)	(2.742)
Household head completed lower secondary (0/1)	0.252*			4.475*	5.496
	(0.140)			(2.490)	(3.356)
Household head is female	−0.166			−2.952	−3.625
	(0.150)			(2.669)	(3.402)
Age of household head	−0.008*	0.129	0.285	0.138	−0.072
	(0.004)	(0.162)	(0.190)	(0.205)	(0.115)
Number of children (0–15 yrs)	0.042			0.742	0.912
	(0.041)			(0.722)	(0.916)
East Sepik Province (base = Bougainville)	−0.745***	−37.029***	−30.890***	−42.419***	−36.927***
	(0.211)	(4.615)	(6.003)	(6.882)	(6.012)
Madang Province (base = Bougainville)	−1.136***	−37.869***	−15.326	−34.059**	− 38.482***
	(0.339)	(6.205)	(14.040)	(15.290)	(7.700)
West Sepik Province (base = Bougainville)	−1.256***	−36.041***	−23.537***	−44.594***	−42.139***
	(0.230)	(5.454)	(9.031)	(9.904)	(6.079)
Inverse Mills (Lambda)			−25.516*		
			(13.969)		
Constant	−2.372***	− 133.379***	−52.404		
	(0.765)	(26.419)	(52.280)		
N Observations	1023	412	1023	1023	1023

Note: ^a^ Major market towns for each area include: Wewak (East Sepik), Maprik (East Sepik), Nuku (West Sepik), Vanimo (West Sepik), Madang (Madang), Kieta (Bougainville), Arawa (Bougainville), Buka (Bougainville). ^b^Not corrected using Heckman procedure using censored sample of non-zero observations of soft drink expenditure

Standard errors in parentheses. ***p < .01, **p < .05, *p < .1

Source: Authors’ calculation using IFPRI PNG-RSFS (2018)

Results from the probit equation reflect expected coefficient results for significant covariates (Table 8). Households with greater total expenditure are associated with a higher probability of purchasing a soft drink. Given that soft drinks are considered a luxury good, households with greater income would have greater flexibility in expenditure decisions. However, and as expected, the probit equation also shows that the probability of purchasing a soft drink decreases as the unit price of a soft drink increases. Results also suggest that as a household head ages, the probability of purchasing a soft drink decreases. This may be that marketing of soft drinks is largely targeted at younger individuals, however further investigation of how marketing campaigns in PNG affect consumer choice behavior is warranted.

The distance to the nearest major market town is negatively correlated with the probability of purchasing a soft drink. The probability of purchasing a soft drink decreases by almost 2% for each 1 km increase in distance to a major market town (and remains significant at the 10% level when we test for joint significance with the quadratic term). Given limited information on availability of soft drinks in each of the sample communities, we use a distance measure to larger market towns, however more precise information on soft drink availability and location could improve this estimate.^12^

When evaluating the second stage Heckman regression results (column C), household expenditure on soft drinks increases until the unit soft drink price reaches about 15 kina per liter, at which point demand decreases. An increase in total household expenditure is also associated with an increase in per capita soft drink consumption (at the 10% significance level), while an increase in the household size is associated with a decrease in per capita consumption. This follows the assumption that consumption is relatively evenly distributed among household members.

Focusing on the unconditional marginal effects, total household expenditure has a strong and positive association with per capita consumption of soft drinks (this holds true for the conditional marginal effects of the censored sample of consumers only). While the price of a soft drink is negatively correlated with the quantity consumed (coefficient of − 6.708), analysis shows that total household income has a quantitatively larger (and positive) association with soft drink consumption. This suggests that policy aimed only at adjusting the price of unhealthy food items may not be enough to curb the increasing demand for ultra-processed food items. Finally, and as expected, results from both conditional and unconditional marginal effects demonstrate that greater distance from a major market is associated with a decrease in the per capita consumption of soft drinks.

## Discussion

The most recent PNG National Nutrition Policy (NNP) published in 2016 outlined a series of objectives and actions to address under- and over-nutrition during the next decade (Government of PNG, 2016 [15]). Policy actions to address over-nutrition included: advocacy and interventions to improve knowledge of and access to nutritionally adequate complementary foods for children; marketing restrictions of high-fat foods and sugar-sweetened beverages; improved food and nutrition labelling that promotes consumer awareness of healthier food and beverage options; and introduction of taxation and subsidy instruments to promote healthy food, while curbing unhealthy food purchases (WHO, 2017 [50]; PNG NNP, 2016 [15]).^13^

The PNG NNP sets forth an ambitious strategy to tackle overnutrition, however the document suggests very little oversight on program indicators or impact, and no clear pathway to policy formation and implementation. The NNP identifies shortcomings in the current governance structure of the nutrition policy (and warns of inertia in documenting evidence of programs and impact during the previous 1995 nutrition policy). Currently, there is no national level organization providing leadership or coordination for addressing nutrition issues. Programs that address nutrition are generally led by individual government departments or development partners that lack coordinated reporting guidelines. Similarly, there is little human capacity to effectively design, develop, and implement programs to address nutrition challenges within country^14^; and PNG lacks baseline data on key nutrition indicators to inform program and policy design.

This paper focuses on the economic drivers (household income, international trade trends, and food prices) of consumption choices, however future research is needed on how marketing of unhealthy foods may be targeting specific populations (younger households) and how advertisement campaigns are associated with consumption outcomes. Garcia-Dorado et al. (2019 [12]) argue that improved terms of trade do not fully explain the increase in ultra-processed foods, but rather foreign direct investment and its links to poor food marketing are also driving unhealthy food consumption in lower middle-income countries. Snowden et al. (2013 [45]) identify the need for legislation and enforcement of good quality food safety and nutrition labelling throughout the Pacific Islands to curb the increasing incidence of non-communicable diseases related to obesity and overnutrition. Thow [47] argues for greater participation of public health nutritionists to inform trade policy decisions to prevent and control diet-related chronic diseases such as diabetes related to obesity and poor diet choices.

Based on the analysis presented here, implementing a tax on sugar-sweetened beverages could induce consumers to substitute soft drink consumption for non-taxed, low sugar alternatives. However, a tax may not be sufficient to curb increased levels of sugar-sweetened beverage consumption. Mixed results of tax policy on unhealthy food items in other contexts suggests a need for further investigation. While Brownell and Frieden [7] report that a 10% increase in the price of soft drinks would lead to an almost 8% decrease in consumption^15^, Wang [49] argues that a similar tax would lead to increased state revenue but not decreased consumption in the United States. Adam and Smed [1] evaluate different soft drink taxation types (e.g., tax based on sugar-added quantity versus flat tax, or tax on container size) to better understand substitution effects among consumer preference, arguing that a tax on sugar content may be the most effective tool to regulate consumption, however warn that these measures are context specific.

Given the limitations of fiscal (tax) policies on consumption decisions, education programs that integrate nutrition and diet information are an important first step in generating awareness of healthy food choices in PNG. A nationally developed and recognized food-based dietary guideline (FBDG) is a good instrument to be used in such education programs to advocate a healthy diet.^16^ Nutrition education programs should not only target primary and secondary age children, but also caretakers and adult populations, female and male, who are the primary decision-makers of household food expenditure and meal preparation. In addition, training of trainers at regional universities as well as training of provincial and district public officials may facilitate nutrition-related activities and information to be integrated into other rural extension services. An important service provider for PNG rural populations are local church-based organizations, who provide a variety of family-based support services, including emergency food aid. Greater awareness and training of local church-based organizations may also provide a platform for greater nutrition-related information dissemination.

This paper focuses on exploring the associates between the consumption of sugar-sweetened drinks and obesity and overweight prevalence in PNG. However, further research is needed on the effects of other food consumption patterns. For example, recent studies have demonstrated how overconsumption of staple foods may to lead to obesity in the other developing countries [17, 26, 40].

Finally, a careful cost comparison of a “business as usual” environment versus a framework that integrates a national and sub-national policy strategy to measure, monitor, and assess the impacts of nutrition-related activities may demonstrate the cost of continued political inertia. Given recent evidence of increasing healthcare costs due to growing non-communicable disease prevalence among overweight and obese populations, poor nutrition may become as costly for governments as it is for individuals suffering from such conditions.

## Conclusion

Recent evidence in the Pacific suggests that increases in economic welfare are straddling many island nations with a double burden of malnutrition, requiring policymakers and development practitioners to address not only food insecurity among vulnerable populations, but also rising overweight and obesity prevalence among those experiencing income gains and greater access to markets (and processed foods). The analysis presented in this paper raises three red flags based on recent data of food consumption and agri-food imports in Papua New Guinea (PNG). First, international import food trade data suggest that PNG imports of unhealthy, ultra-processed sugar-sweetened beverages and food have increased substantially over time. The largest increase in value of overall processed food imports between 2001-05 and 2014–18 was dominated by sugar-sweetened beverages (accounting for 16% of the total processed imports in 2014–18, and increasing by 23% per capita per year between 2001 and 2018). Second, households across PNG with a greater food expenditure share on sugar-sweetened beverages have a higher probability of an overweight child (under 5 years old), controlling for other household characteristics. Last, the probability and prevalence of soft-drink consumption in PNG increases with greater income acquisition and improved market access. While the price of a soft drink is negatively correlated with the quantity consumed, analysis suggests that total household income has a quantitatively larger (and positive) association with soft drink consumption.

Several recent initiatives, financed by international donors, aimed at improving nutrition outcomes are being piloted in different areas of the country. For example, the Morobe Revitalizing Agriculture and Nutrition in the Education System project uses a school gardens approach to provide information on improved agriculture processes and dietary diversity in primary and secondary schools. The Eat Smart campaign aims to promote healthier consumption choices through televised cooking shows, radio broadcasts and interactive cooking sessions with school aged children. While international donors, non-governmental organizations, and private sector actors have advocated for and funded development projects that promote healthier eating habits, there is little evidence as to how these projects are affecting overall nutrition outcomes in PNG. A concerted effort to evaluate these programs could inform future policy initiatives and investments aimed at improving nutrition indicators in PNG.

While increases in household income and improved rural-urban linkages are crucial for fostering economic growth and transformation, a portfolio of health and nutrition education, advocacy and policy is needed to ensure that PNG’s economic transition is dovetailed with improved household health and wellness outcomes. Education programs and information campaigns to inform households of the tradeoffs of consuming less healthy processed foods will also be necessary to address the substantial increase in demand of these products. Given the lack of trained nutritionists in PNG, a concerted effort and financial investment in training healthcare workers and public servants will be necessary to ensure policy is met with action to achieve improved nutrition across the country.

## Supplementary Information

**Additional file 1.** Appendix Table 1: Share of ultra-processed food and processed food imports. Appendix Table 2: HIES descriptive statistics of covariates included in probit regression on obesity/overweight. Appendix Table 3: Probit regression on associates of household with an obese/overweight child (under 5). Appendix Table 4: Descriptive statistics of covariates included in Heckman model. Appendix Table 5: Sensitivity of exclusion restriction of Heckman model for soft drink expenditure per capita. Appendix Table 6: Heckman sample selection model for soft drink expenditure per capita using HIES (2009/10).

## Data Availability

The primary datasets generated and/or analysed during the current study are available in the Harvard dataverse repository, https://dataverse.harvard.edu/dataset.xhtml?persistentId=doi:10.7910/DVN/ZXRD6N. Secondary datasets analysed for this study (HIES 2009/10 and the BACI trade database) are available at their respective sources cited in the references.

## Footnotes

1 Poskitt (2014) provides evidence from a range of low and lower-middle income countries across the world who’s overweight and obesity prevalence is increasing. Similarly, the World Health Organization (2017 [50]) published an in-depth study showing substantial increases of overweight and obesity prevalence in the West Pacific region.

2 This allows for a smoothing of yearly import fluctuations and inconsistencies in the import and export data reported by country and compiled within the BACI dataset.

3 A variety of studies evaluating protein consumption and dietary diversity have linked stunting rates to protein deficiency and lack of essential amino acids in young children [11, 14, 43].

4 Specific items, which are not, by definition, considered ultra-processed (e.g. butter, cheese, and sweetened milk), are included in the sugar-sweetened food and beverage, and high-saturated fat food groups.

5 This share includes several items which, by definition, are not ultra-processed (e.g. butter, cheese, and sweetened milk) given these item’s high- sugar or saturated fat content.

6 Given the rural nature of the survey, the sample size of overweight or obese individuals is not large enough for robust analysis.

7 Children are classified as overweight, and obese if their weight-for-height is 2 and 3 standard deviations above WHO Child Growth Standards, respectively.

8 For evaluations of the Heckman two-stage approach in household budget studies, see McDonald and Moffitt [29]; Heien and Wesseils [19]; Hoffman and Kassouf (2005 [21]); Adusah-Poku and Takeuchi [2].

9 Results comparing households with at least one obese child should be evaluated with caution given the small sample size in both non-poor and poor households.

10 While the 2018 RSFS survey was not designed to be representative at a specific administrative level, the spread of the 2018 sample (including Nuku district in West Sepik, Maprik and surrounding districts in East Sepik, and Middle Ramu and surrounding districts in Madang) provides wide variation in household livelihood structure and market access within Momase region.

11 These results echo other studies that evaluate soft drink consumption with obesity rates among children and adults, including a recent study by Kessaram et al. [25] focusing on Pacific Island youth (other studies include Beck et al. [6] and Ludwig et al. [28].

12 Gibson and Rozelle [13] used a detailed rural road dataset and travel time analysis to show that improved rural road access has a significant and positive impact on household income, particularly in poor households, in Papua New Guinea.

13 As of 2016, only about 1/3 of countries with data on obesity and overweight prevalence were on track to meet the childhood overweight targets by 2025 set by the World Health Assembly (WHO, 2017).

14 According to the PNG NNP (2016) there has been no pre-service or in-service training in nutrition for more than 20 years in PNG.

15 Similar results on effectiveness of decreasing sweetened beverage consumption (and associated health risks) via a sales tax include Smith et al. [44] and Nielsen and Popkin [32].

16 Approximately 100 countries worldwide have developed an FBDG. Thus far, the PNG government has not developed a national FBDG, however a variety of countries in the region have an FBDG including Indonesia, Fiji, Philippines, and Australia (https://www.fao.org/nutrition/education/food-dietary-guidelines/regions/asia-pacific/en/).
