# Supplementary material for: Papua New Guinea agri-food trade and household consumption trends point towards dietary change and increased overweight and obesity prevalence
Source: Global Health. 2021 Nov 27;17:135. doi: 10.1186/s12992-021-00787-0 (PMC8626949; doi:10.1186/s12992-021-00787-0)
Supplement: Supplementary file 1 — Additional file 1. Appendix Table 1: Share of ultra-processed food and processed food imports. Appendix Table 2: HIES descriptive statistics of covariates included in probit regression on obesity/overweight. Appendix Table 3: Probit regression on associates of household with an obese/overweight child (under 5). Appendix Table 4: Descriptive statistics of covariates included in Heckman model. Appendix Table 5: Sensitivity of exclusion restriction of Heckman model for soft drink expenditure per capita. Appendix Table 6: Heckman sample selection model for soft drink expenditure per capita using HIES (2009/10). [file 12992_2021_787_MOESM1_ESM.docx]

**Appendix Table 1: Share of ultra-processed food and processed food imports**

| Food category and type | Share in ultra-processed food imports | | | Share in total processed food imports | | |
| --- | --- | --- | --- | --- | --- | --- |
|  | 2001/05 | 2014/18 | Difference | 2001/05 | 2014/18 | Difference |
| Sugar-sweetened food |  |  |  |  |  |  |
| Sugar confectionery | 7.6% | 5.2% | -2.4% | 4.7% | 3.6% | -1.0% |
| Malt extract | 6.7% | 5.1% | -1.6% | 4.1% | 3.5% | -0.6% |
| Chocolate | 2.3% | 1.3% | -1.0% | 1.4% | 0.9% | -0.5% |
| Jams, fruit puree | 0.6% | 0.6% | 0.0% | 0.4% | 0.4% | 0.1% |
| Total sugar-sweetened food | 17.2% | 12.1% | -5.0% | 10.5% | 8.5% | -2.1% |
| Sugar-sweetened drinks |  |  |  |  |  |  |
| Non-alcoholic drinks (including soft drinks) | 6.2% | 22.4% | 16.2% | 3.8% | 15.6% | 11.8% |
| Coffee concentrate, tea or mate | 2.2% | 2.7% | 0.5% | 1.3% | 1.9% | 0.5% |
| Fruit/ vegetable juices | 5.4% | 1.1% | -4.3% | 3.3% | 0.8% | -2.6% |
| Sweetened whey products |  |  |  | 0.1% | 0.1% | 0.0% |
| Sweetened buttermilk, cream, yoghurt |  |  |  | 1.7% | 1.1% | -0.6% |
| Flavored/sweetened milk |  |  |  | 7.2% | 1.8% | -5.4% |
| Total sugar-sweetened drinks | 13.9% | 26.2% | 12.3% | 17.5% | 21.2% | 3.7% |
| High saturated fat foods |  |  |  |  |  |  |
| Food preparations | 21.1% | 31.0% | 9.9% | 12.9% | 21.6% | 8.6% |
| Prepared/preserved meat | 10.3% | 6.1% | -4.3% | 6.3% | 4.2% | -2.1% |
| Pastry, cakes, biscuits | 4.3% | 5.5% | 1.2% | 2.7% | 3.8% | 1.2% |
| Margarine | 10.3% | 3.1% | -7.1% | 6.3% | 2.2% | -4.1% |
| Ice cream | 0.9% | 0.7% | -0.2% | 0.6% | 0.5% | -0.1% |
| Sausages and products | 0.3% | 0.7% | 0.4% | 0.2% | 0.5% | 0.3% |
| Potato chips | 2.0% | 0.5% | -1.5% | 1.2% | 0.3% | -0.9% |
| Cocoa Butter, fat and oil |  |  |  | 0.0% | 0.0% | 0.0% |
| Butter |  |  |  | 3.6% | 1.0% | -2.6% |
| Cheese and curd |  |  |  | 2.1% | 0.8% | -1.2% |
| Total high saturated fat foods | 49.2% | 47.6% | -1.6% | 35.9% | 35.0% | -0.9% |
| Other ultra-processed |  |  |  |  |  |  |
| Pasta | 2.3% | 6.6% | 4.2% | 1.4% | 4.6% | 3.2% |
| Sauces and preparations | 10.0% | 4.1% | -5.9% | 6.1% | 2.9% | -3.3% |
| Soups and broths | 2.2% | 1.6% | -0.6% | 1.3% | 1.1% | -0.3% |
| Yeasts | 3.5% | 1.0% | -2.5% | 2.2% | 0.7% | -1.4% |
| Cereal products | 1.6% | 0.7% | -0.9% | 1.0% | 0.5% | -0.5% |
| Vinegar | 0.1% | 0.1% | 0.0% | 0.0% | 0.1% | 0.0% |
| Tapioca | 0.0% | 0.0% | 0.0% | 0.0% | 0.0% | 0.0% |
| Coffee mate | 0.0% | 0.0% | 0.0% | 0.0% | 0.0% | 0.0% |
| Albumins | 0.0% | 0.0% | 0.0% | 0.0% | 0.0% | 0.0% |
| Total other ultra-processed | 19.7% | 14.1% | -5.6% | 12.1% | 9.8% | -2.3% |
| Total shares | 100.0% | 100.0% |  | 76.0% | 74.5% |  |

Note: A few processed items are not ultra-processed, so their share in ultra-processed are missing in the above table.

Source: Authors’ calculation using BACI (2021).

**Appendix Table 2: HIES descriptive statistics of covariates included in probit regression on obesity/overweight**

|  | N | mean | sd | min | max |
| --- | --- | --- | --- | --- | --- |
| Obese (0/1) | 1721 | 0.07 | 0.26 | 0 | 1 |
| Overweight (0/1) | 1721 | 0.15 | 0.36 | 0 | 1 |
| Share of consumption of sugar-sweetened food in total food (%) | 1721 | 0.01 | 0.02 | 0.00 | 0.31 |
| Share of consumption of sugar-sweetened beverages in total food (%) | 1721 | 0.04 | 0.06 | 0.00 | 0.39 |
| Share of consumption of high-saturated fat food in total food (%) | 1721 | 0.05 | 0.07 | 0.00 | 0.62 |
| Total HH expenditure (PGK/capita/year/1000) | 1721 | 2.20 | 2.01 | 0.04 | 24.05 |
| Household in metro area (0/1) | 1721 | 0.20 | 0.40 | 0 | 1 |
| Household in urban area (0/1) |  | 0.47 | 0.50 | 0 | 1 |
| Number of children (0-15) | 1721 | 3.07 | 1.58 | 1 | 11 |
| Household size | 1721 | 6.66 | 2.76 | 2 | 20 |
| Female Household head (0/1) | 1721 | 0.13 | 0.33 | 0 | 1 |
| Age of Household head | 1721 | 40.65 | 11.48 | 17 | 89 |
| Household head completed primary education (0/1) | 1721 | 0.30 | 0.46 | 0 | 1 |
| Southern (0/1) | 1721 | 0.34 | 0.47 | 0 | 1 |
| Highland region (0/1) | 1721 | 0.20 | 0.40 | 0 | 1 |
| Momase region (0/1) | 1721 | 0.32 | 0.47 | 0 | 1 |
| Island region (0/1) | 1721 | 0.14 | 0.35 | 0 | 1 |

Note: Overweight includes obese.

Source: Authors’ calculation using HIES (2009/10).

**Appendix Table 3: Probit regression on associates of household with an obese/overweight child (under 5)**

| **Dependent variable: Household with at least one obese/overweight child (under 5)** | **Obese child** | | **Overweight child** | |
| --- | --- | --- | --- | --- |
|  | **All regions** | **Momase region** | **All regions** | **Momase region** |
|  |  |  |  |  |
| Share of consumption of sugar-sweetened food in total food (%) | 1.581 | 3.224 | 0.426 | 5.657 |
|  | (3.454) | (5.472) | (2.750) | (4.533) |
| Share of consumption of sugar-sweetened beverages in total food (%) | 1.201 | 7.494* | 2.152** | 6.605** |
|  | (1.405) | (3.885) | (0.918) | (2.778) |
| Share of consumption of high-saturated fat food in total food (%) | 0.436 | -4.426 | 0.315 | -0.068 |
|  | (1.170) | (3.269) | (1.027) | (1.962) |
| Total HH expenditure, thousands (PGK/capita/year) | -0.015 | 0.025 | -0.044 | -0.017 |
|  | (0.034) | (0.045) | (0.032) | (0.042) |
| Household in metro area (0/1) | 0.218 | 0.213 | 0.105 | 0.094 |
|  | (0.241) | (0.369) | (0.161) | (0.295) |
| Household in urban area (0/1) | -0.211 | -0.342 | -0.163 | -0.448 |
|  | (0.129) | (0.460) | (0.110) | (0.339) |
| Number of children (0-15) | 0.008 | 0.071 | 0.044 | 0.168*** |
|  | (0.050) | (0.088) | (0.039) | (0.061) |
| Household size | 0.045 | 0.062 | 0.052** | 0.022 |
|  | (0.032) | (0.054) | (0.021) | (0.046) |
| Female Household head (0/1) | -0.118 | -0.031 | 0.143 | -0.507 |
|  | (0.188) | (0.508) | (0.223) | (0.445) |
| Age of Household head | -0.006 | -0.010 | -0.008* | -0.001 |
|  | (0.005) | (0.008) | (0.004) | (0.007) |
| Household head completed primary education (0/1) | 0.047 | -0.228 | 0.113 | -0.109 |
|  | (0.134) | (0.313) | (0.094) | (0.285) |
| Highland region (base = Southern) | 0.553** |  | 0.722*** |  |
|  | (0.220) |  | (0.159) |  |
| Momase region (base = Southern) | -0.041 |  | -0.075 |  |
|  | (0.236) |  | (0.151) |  |
| Island region (base = Southern) | 0.137 |  | -0.080 |  |
|  | (0.287) |  | (0.259) |  |
| Pseudo R2 | 0.045 | 0.082 | 0.086 | 0.089 |
| N Observations | 1,721 | 544 | 1,721 | 544 |

Source: Authors’ calculation using HIES (2009/10). Note: Probit regression coefficient is reported. Robust standard errors clustered by census unit in parentheses. *** p < 0.01. ** p < 0.05. * p < 0.10.

**Appendix Table 4: Descriptive statistics of covariates included in Heckman model**

|  | N | Mean | SD | Min | Max |
| --- | --- | --- | --- | --- | --- |
| Expenditure on soft drink per capita per year | 1023 | 18.91 | 34.78 | 0 | 263.28 |
| Log of total household expenditure (PGK/capita/year) | 1023 | 7.36 | 0.68 | 3.95 | 9.3 |
| Household-level unit soft drink price (PGK/liter) | 1023 | 8.17 | 2.75 | 0.29 | 15.8 |
| Euclidean distance to major market town^a^ (km) | 1023 | 54.83 | 48.95 | 0.59 | 140.81 |
| Household size | 1023 | 5.91 | 2.21 | 1 | 17 |
| Household head completed primary education (0/1) | 1023 | 0.38 | 0.48 | 0 | 1 |
| Household head completed low secondary education (0/1) | 1023 | 0.22 | 0.42 | 0 | 1 |
| Household head is female | 1023 | 0.1 | 0.3 | 0 | 1 |
| Age of household head (years) | 1023 | 41.9 | 11.93 | 16 | 88 |
| Number of children (0-15 years old) | 1023 | 2.71 | 1.73 | 0 | 9 |
| Bougainville (0/1) | 1023 | 0.24 | 0.43 | 0 | 1 |
| East Sepik (0/1) | 1023 | 0.24 | 0.43 | 0 | 1 |
| Madang (0/1) | 1023 | 0.29 | 0.45 | 0 | 1 |
| West Sepik (0/1) | 1023 | 0.23 | 0.42 | 0 | 1 |

Note: PGK = Papua New Guinea Kina

^a^ Major market town for each area include: Wewak (East Sepik), Maprik (East Sepik), Nuku (West Sepik), Vanimo (West Sepik), Madang (Madang), Kieta (Bougainville), Arawa (Bougainville), Buka (Bougainville); USD 1.00 = PGK 3.28 in June 2018.

Source: Authors’ calculation using IFPRI PNG-RSFS (2018).

**Appendix Table 5: Sensitivity of exclusion restriction of Heckman model for soft drink expenditure per capita**

|  | Specification 1 | | | | | Specification 2 | | | | | Specification 3 | | | | |
| --- | --- | --- | --- | --- | --- | --- | --- | --- | --- | --- | --- | --- | --- | --- | --- |
| Dependent variables:  Expenditure on soft drink per capita per year | Participation equation (Probit) | Consumption equation | | Marginal effects | | Participation equation (Probit) | Consumption equation | | Marginal effects | |  | Consumption equation | | Marginal effects | |
|  |  | Without correction^b^ | Heckman procedure | Conditional marginal effects | Unconditional marginal effects |  | Without correction^b^ | Heckman procedure | Conditional marginal effects | Unconditional marginal effects | Participation equation (Probit) | Without correction^b^ | Heckman procedure | Conditional marginal effects | Unconditional marginal effects |
|  | A | B | C | D | E | A | B | C | D | E | A | B | C | D | E |
| Log of total household expenditure (PGK/capita/year) | 0.673*** | 24.018*** | 4.561 | 24.860*** | 19.691*** | 0.673*** | 22.101*** | 10.585 | 23.389** | 19.008*** | 0.673*** | 21.961*** | 10.159 | 23.570** | 19.084*** |
|  | (0.077) | (3.139) | (8.636) | (8.953) | (2.922) | (0.077) | (3.191) | (9.087) | (9.208) | (2.923) | (0.077) | (3.242) | (9.405) | (9.534) | (3.077) |
| Household-level unit soft drink price (PGK/kg) | -0.461*** | 5.797*** | 16.180*** | 2.268 | -6.130** | -0.461*** | 5.973*** | 12.465** | 3.689 | -5.503** | -0.461*** | 6.024*** | 12.729** | 3.537 | -5.567** |
|  | (0.078) | (2.115) | (5.009) | (5.531) | (2.764) | (0.078) | (2.104) | (5.304) | (5.506) | (2.624) | (0.078) | (2.116) | (5.515) | (5.729) | (2.731) |
| Household-level unit soft drink price squared (PGK/kg) | 0.025*** | -0.181 | -0.738*** | 0.006 | 0.376** | 0.025*** | -0.179 | -0.532* | -0.063 | 0.346** | 0.025*** | -0.182 | -0.546* | -0.054 | 0.349** |
|  | (0.004) | (0.123) | (0.273) | (0.300) | (0.148) | (0.004) | (0.122) | (0.291) | (0.302) | (0.140) | (0.004) | (0.123) | (0.302) | (0.313) | (0.146) |
| Euclidean distance to major market town^a^ (km) | -0.014** |  |  | -0.410** | -0.363* | -0.014** |  |  | -0.259** | -0.303 | -0.014** |  |  | -0.271** | -0.307 |
|  | (0.007) |  |  | (0.206) | (0.205) | (0.007) |  |  | (0.130) | (0.185) | (0.007) |  |  | (0.136) | (0.191) |
| Euclidean distance to major market town squared (km^2^) | 0.000 |  |  | 0.002 | 0.001 | 0.000 |  |  | 0.001 | 0.001 | 0.000 |  |  | 0.001 | 0.001 |
|  | (0.000) |  |  | (0.001) | (0.001) | (0.000) |  |  | (0.001) | (0.001) | (0.000) |  |  | (0.001) | (0.001) |
| Household size | 0.068** | -1.262 | -3.004* | -0.939 | 0.680 | 0.068** | -1.020 | -2.219 | -0.916 | 0.676 | 0.068** | -1.047 | -2.253 | -0.889 | 0.687 |
|  | (0.033) | (1.267) | (1.641) | (1.914) | (1.056) | (0.033) | (1.263) | (1.606) | (1.722) | (0.909) | (0.033) | (1.269) | (1.625) | (1.750) | (0.927) |
| Household head completed primary education (0/1) | 0.075 |  |  | 2.275 | 2.012 | 0.075 | 1.252 | 0.345 | 1.779 | 1.809 | 0.075 | 1.295 | 0.265 | 1.768 | 1.805 |
|  | (0.124) |  |  | (3.748) | (3.353) | (0.124) | (4.908) | (5.259) | (5.766) | (3.418) | (0.124) | (4.916) | (5.309) | (5.858) | (3.462) |
| Household head completed lower secondary (0/1) | 0.252* |  |  | 7.600* | 6.721 | 0.252* | 10.070* | 6.505 | 11.299* | 8.086** | 0.252* | 10.049* | 6.355 | 11.376* | 8.118** |
|  | (0.140) |  |  | (4.227) | (4.120) | (0.140) | (5.192) | (6.149) | (6.702) | (3.906) | (0.140) | (5.199) | (6.239) | (6.835) | (3.973) |
| Household head is female | -0.170 |  |  | -5.122 | -4.529 | -0.170 |  |  | -3.231 | -3.777 | -0.170 | -1.660 | 1.421 | -1.963 | -3.295 |
|  | (0.150) |  |  | (4.527) | (4.168) | (0.150) |  |  | (2.855) | (3.591) | (0.150) | (6.522) | (7.194) | (7.791) | (4.316) |
| Age of household head | -0.008* | 0.118 | 0.347 | 0.093 | -0.092 | -0.008* | 0.139 | 0.274 | 0.114 | -0.083 | -0.008* | 0.140 | 0.279 | 0.111 | -0.084 |
|  | (0.004) | (0.176) | (0.224) | (0.259) | (0.140) | (0.004) | (0.175) | (0.211) | (0.226) | (0.122) | (0.004) | (0.176) | (0.214) | (0.230) | (0.124) |
| Number of children (0-15 years old) | 0.041 | -0.367 | -1.398 | -0.173 | 0.550 | 0.041 | -0.527 | -1.115 | -0.342 | 0.478 | 0.041 | -0.544 | -1.128 | -0.319 | 0.488 |
|  | (0.041) | (1.643) | (1.952) | (2.304) | (1.310) | (0.041) | (1.631) | (1.782) | (1.943) | (1.129) | (0.041) | (1.634) | (1.796) | (1.970) | (1.145) |
| East Sepik Province  (base = Bougainville) | -0.744*** | -36.618*** | -25.733*** | -45.283*** | -38.042*** | -0.744*** | -34.524*** | -28.506*** | -40.838*** | -35.758*** | -0.744*** | -34.501*** | -28.240*** | -41.157*** | -35.924*** |
|  | (0.211) | (4.625) | (7.099) | (9.116) | (7.111) | (0.211) | (4.669) | (6.708) | (7.616) | (6.219) | (0.211) | (4.675) | (6.909) | (7.874) | (6.326) |
| Madang Province  (base = Bougainville) | -1.132*** | -36.912*** | 2.704 | -28.976 | -37.111*** | -1.132*** | -35.940*** | -11.337 | -31.321 | -36.974*** | -1.132*** | -35.886*** | -10.217 | -31.148 | -36.986*** |
|  | (0.338) | (6.247) | (17.645) | (20.416) | (9.535) | (0.338) | (6.210) | (19.185) | (20.249) | (7.877) | (0.338) | (6.221) | (20.124) | (21.237) | (8.011) |
| West Sepik Province  (base = Bougainville) | -1.259*** | -35.112*** | -12.567 | -48.417*** | -42.997*** | -1.259*** | -34.283*** | -20.401* | -43.015*** | -40.982*** | -1.259*** | -34.227*** | -19.790 | -43.477*** | -41.147*** |
|  | (0.230) | (5.485) | (11.178) | (13.139) | (7.113) | (0.230) | (5.455) | (11.719) | (12.503) | (6.547) | (0.230) | (5.466) | (12.221) | (13.045) | (6.716) |
| Inverse Mills (Lambda) |  |  | -43.277** |  |  |  |  | -27.299 |  |  |  |  | -28.594 |  |  |
|  |  |  | (17.306) |  |  |  |  | (19.878) |  |  |  |  | (21.022) |  |  |
| Constant | -2.284*** | -140.936*** | -13.187 |  |  | -2.284*** | -134.565*** | -56.415 |  |  | -2.284*** | -133.473*** | -53.644 |  |  |
|  | (0.758) | (27.491) | (60.350) |  |  | (0.758) | (27.374) | (64.097) |  |  | (0.758) | (27.740) | (65.989) |  |  |
| N Observations | 0.673*** | 24.018*** | 4.561 | 24.860*** | 19.691*** | 0.673*** | 22.101*** | 10.585 | 23.389** | 19.008*** | 0.673*** | 21.961*** | 10.159 | 23.570** | 19.084*** |

Source: Authors’ calculation using IFPRI PNG-RSFS (2018). Robust standard errors in parentheses. *** p < 0.01. ** p < 0.05. * p < 0.10.

**Appendix Table 6: Heckman sample selection model for soft drink expenditure per capita using HIES (2009/10)**

| Dependent variables:  Expenditure on soft drink per capita per year | Participation equation (Probit) | Consumption equation | | Marginal effects | |
| --- | --- | --- | --- | --- | --- |
|  |  | Without correction^a^ | Heckman procedure | Conditional marginal effects | Unconditional marginal effects |
|  | A | B | C | D | E |
| Log of total household expenditure (PGK/capita/year) | 0.818*** | 50.257*** | 41.913*** | 51.093*** | 45.629*** |
|  | (0.057) | (3.297) | (5.269) | (5.310) | (2.362) |
| Household-level unit soft drink price (PGK/kg) | -0.258*** | 6.098 | 8.626 | 5.735 | -1.390 |
|  | (0.093) | (5.352) | (5.481) | (5.579) | (3.957) |
| Household-level unit soft drink price squared (PGK/kg) | 0.008* | -0.362 | -0.439 | -0.351 | -0.064 |
|  | (0.004) | (0.311) | (0.312) | (0.316) | (0.214) |
| Urban (0/1) | 0.884*** |  |  | 9.909*** | 22.346*** |
|  | (0.083) |  |  | (0.930) | (3.176) |
| Household size | 0.117*** | -0.738 | -1.739* | -0.426 | 1.926*** |
|  | (0.016) | (0.779) | (0.935) | (0.952) | (0.610) |
| Household head completed primary education (0/1) | -0.133 |  |  | -1.488 | -3.355 |
|  | (0.082) |  |  | (0.922) | (2.111) |
| Household head is female | 0.016 |  |  | 0.174 | 0.393 |
|  | (0.110) |  |  | (1.235) | (2.785) |
| Age of household head | 0.003 | 0.007 | -0.058 | -0.020 | 0.050 |
|  | (0.003) | (0.190) | (0.192) | (0.196) | (0.143) |
| Number of children (0-15 years old) | -0.006** |  |  | -0.064** | -0.144** |
|  | (0.002) |  |  | (0.028) | (0.064) |
| Highland region (base = Southern) | 0.492*** | -10.545* | -12.082** | -7.181 | 4.409 |
|  | (0.112) | (6.022) | (6.072) | (6.170) | (5.102) |
| Momase region (base = Southern) | -0.375*** | -16.493*** | -14.767*** | -19.286*** | -17.143*** |
|  | (0.096) | (5.310) | (5.466) | (5.586) | (3.706) |
| Island region (base = Southern) | 0.120 | -16.530** | -15.466** | -14.158** | -6.454 |
|  | (0.119) | (6.937) | (6.966) | (7.085) | (5.338) |
| Inverse Mills (Lambda) |  |  | -19.301** |  |  |
|  |  |  | (9.839) |  |  |
| Constant | -5.329*** | -331.362*** | -262.899*** |  |  |
|  | (0.685) | (36.072) | (49.607) |  |  |
| N Observations | 1,721 | 976 | 1,721 | 1,721 | 1,721 |

Note: ^a^ Not corrected using Heckman procedure using censored sample of non-zero observations of soft drink expenditure.

Standard errors in parentheses. ***p < .01, **p < .05, *p < .10

Source: Authors’ calculation using HIES 2009/10.
